# A systematic review and meta-analysis of laparotomy compared with laparoscopic management of interstitial pregnancy

**Published:** 2021-01-08

**Authors:** G Marchand, A Taher Masoud, K Sainz, A Azadi, K Ware, J Vallejo, S Anderson, A King, A Osborn, S Ruther, G Brazil, K Cieminski, S Hopewell, L Rials, D Jenks, A Steele, J Love

**Affiliations:** Marchand Institute for Minimally Invasive Surgery, Mesa, Arizona, USA;; Faculty of Medicine, Fayoum University, Fayoum, Egypt; Star Urogynecology, Department of Urogynecology, Peoria, Arizona, USA; Midwestern University College of Osteopathic Medicine, Glendale, Arizona, USA

**Keywords:** Interstitial pregnancy, cornual, ectopic, laparoscopy, laparotomy, meta-analysis

## Abstract

**Background:**

Interstitial pregnancy is a rare but life-threatening condition accounting for 1-4% of all types of tubal ectopic pregnancies. It can be managed by open and minimally invasive surgical techniques. Our goal was to compare laparoscopic and open surgery for managing interstitial pregnancy.

**Search Strategy:**

We searched PubMed, Scopus, Web of Science, and Cochrane up to May 2020.

**Selection Criteria:**

1) Women with interstitial pregnancy, 2) Intervention: laparoscopic surgery, 3) Comparator: open surgery, 4) Outcomes: Hospital stay, operation time, pain scale, blood loss. Secondary outcomes: any other reported 5) Study designs: interventional and observational.

**Data collection and analysis:**

Data was extracted from the relevant articles and was pooled as mean difference (MD) or relative risk (RR) with a 95% confidence interval (CI).

**Main Results:**

We included six studies, three of which provided eligible data. The duration of hospital stay was lower in the laparoscopic surgery group (MD = -1.42, 95% CI [-1.72, -0.76], P < 0.0001). There was no significant difference in operative time (MD = 5.90, 95% CI [-11.30, 23.09], P = 0.50, blood loss (MD = -9.43, 95% CI [-214.18, 195.32], P = 0.93), complications (RR = 1.54, 95% CI [0.20, 11.85], P = 0.68), or blood transfusions (RR = 0.77, 95% CI [0.50, 1.25], P = 0.30).

**Conclusion:**

Laparoscopic surgery is associated with shorter hospital stay, with no difference in terms of blood loss, post-, and intraoperative complications, and need for blood transfusion compared with laparotomy.

## Introduction

Interstitial pregnancy is a type of ectopic pregnancy which occurs in the uterus but outside the uterine cavity, where implantation occurs in the interstitial (proximal) part of the fallopian tube at its insertion into the uterus (Moawad et al., 2010; Wai-man et al., 2001; Malinowski et al., 2006). It is a life- threatening rare condition with an incidence of about 1-4% of all types of tubal ectopic pregnancies and approximately one for every 2500-5000 live births (Rock et al., 2008). Many risk factors may predispose to interstitial pregnancy, including pelvic surgery and inflammatory diseases, tumours, anomalies of the uterus, and in-vitro fertilisation (Lau et al., 1999). Many cases can be asymptomatic or present with non-specific symptoms such as vaginal bleeding and abdominal pain; therefore diagnosis is often delayed which increases the risk of rupture (Lau et al., 1999; Habana et al., 2000; Soriano et al., 2008). Diagnosis depends on high levels of suspicion, especially in women who have any of the above risk factors. Interstitial and cornual pregnancy may be used as synonyms of each other as reported in some studies, however, the term interstitial pregnancy will be used in this text to avoid confusion with intrauterine pregnancies in one horn of a bicornuate uterus. Different definitions of cornual pregnancy are described in the literature, some of which differentiate these terms or use them as synonyms (Habana et al., 2000; Soriano et al., 2008; Faraj et al., 2007; BJOG Greentop Guideline #21, 2016).

Many modalities were used for management including medical treatment such as local and systemic methotrexate, expectant management, open and minimally invasive surgical techniques (Moawad et al., 2010). Choosing a treatment option is dependent on criteria such as the patient’s desire for future fertility and whether the rupture has occurred or not. Medical or expectant treatment are used only in asymptomatic and hemodynamically stable patient cases (Moawad et al., 2010). Surgical management is the main line of management in most cases, especially in ruptured ones. These surgical options are either laparoscopy or laparotomy depending on the patient’s condition and available resources. Traditionally, open surgery such as laparotomy with cornual resection or hysterectomy was used, but with the progression in surgical approaches, laparoscopic surgeries have shown better results compared with laparotomy (Ng et al., 2009). Laparoscopic surgery for ectopic pregnancies can be performed through different approaches, such as cornuostomy, salpingostomy, cornual resection, and mini-cornual excision (Sagiv et al., 2001; Chan et al., 2003; Tulandi et al., 1995; Vilos et al., 1995; Bremner et al., 2000). Laparoscopic surgery may have advantages over laparotomy, such as lower hospital stay duration, less postoperative pain, lower blood loss, and skin incision (Ehrenberg- Buchner et al., 2009; Marchand 2019).

Some reports have reviewed most of the treatment modalities, and described a road map for the management of ectopic pregnancies, but these reports include few or no interstitial pregnancy cases (Moawad et al., 2010; Cucinella et al., 2014) and show no clear evidence for selecting the most suitable surgical approach in interstitial pregnancy. Therefore, we aim to compare laparotomy with laparoscopic management of interstitial pregnancy, as evidenced from published studies.

## Methods

This systematic review and meta-analysis was conducted following the Preferred Reporting Items for Systematic Reviews and Meta-Analyses (PRISMA) statement (Moher et al., 2009). We also followed the guidelines reported in the Cochrane Handbook for Systematic Reviews of Interventions (Higgins et al., 2008).

## Literature Search

We searched for published studies in four electronic databases: PubMed, Web of Science, Scopus, and Cochrane Central Register of Controlled Trials (CENTRAL) in June 2020. We used the following query for our search: ((Laparoscop* OR cornuostomy) AND (cornu* OR laparotomy OR “cornual evacuation” OR “cornual resection” OR “cornual excision” OR “wedge resection” OR “loop ligature” OR “Vicryl loop placement” OR “conical exeresis” OR hysterectomy OR salping* OR traditional OR classic* OR conventional)) AND (“interstitial pregnancy” OR “Cornual pregnancy” OR “cornual gestation” OR “interstitial gestation” OR “cornual ectopic”).

## Eligibility criteria

We included all studies that met the following criteria: 1) Patients: women with interstitial (cornual) pregnancy, 2) Intervention: all types of laparoscopic surgeries, 3) Comparator: all types of open surgeries, 4) Outcomes: main outcomes included; hospital stay, operation time, pain on VAS scale, and blood loss. Secondary outcomes; any other reported outcome, and 5) Study design: all interventional and observational studies (Cohort, case-control, cross- sectional, case series and case report). We excluded conference abstracts, non-English language studies, reviews, and studies that report the effect of only one type of surgery. No restriction was placed on age, place, and publication date.

## Screening and studies selection

Two independent authors (KW and GB) screened the search results for eligibility in two steps: title and abstracts were screened, then full-text screening occurred in which the articles were revised for all criteria to be included in our study. A third author (KS) resolved any dispute that occurred. We manually screened the references of the included studies, and previous systematic review, for additional or missed citations.

## Data Extraction

After screening , two authors (AK and SH) independently extracted the following data from the eligible studies using a previous formatted data extraction sheet: (1) Summary of the included studies including study design, sites and time, participants and main inclusion criteria, total sample size, type of laparoscopic surgery, type of traditional surgery, number of patients assigned to each type, investigations, results, conclusion of each study; (2) baseline characteristics of the patients in each study including groups, cases numbers in each group, age, gestational age (days), number of symptomatic women at diagnosis, number of ruptured ectopic, parity, gravidity, and, risk factors for developing interstitial pregnancy in recruited patients; (3) any repeated outcomes (reported by two or more studies) including postoperative hospital stay (days), operative time (minutes), blood loss (ml), post- and intraoperative complications, need for blood transfusion. Data for continuous outcomes were extracted as a mean and standard deviation, and dichotomous outcomes, events and total were extracted.

## Quality assessment

The quality of the included studies were assessed by quality assessment tools of the National Heart, Lung, and Blood Institute (NHLBI) (NIH Quality Assessment Tools, 2020). We used a tool for observational cohort studies and another tool for a case series study. Each tool was composed of some questions to assess the risk of bias and confounders. These questions were answered by “yes”, “no”, “not applicable”, “cannot determine”, or “not reported”, with each study then being given a score to guide the overall rating of the quality as “good”, “fair”, or “poor”. We could not assess the publication bias due to the small number of included studies according to Egger’s funnel-plot-based methodologies (Egger et al., 1997).

## Data Synthesis

For continuous data, we used the inverse-variance method and the data was pooled as mean difference (MD) using Review Manager Software (version 5.3) for Windows. For dichotomous data, we used the Mantel-Hanszel method and the data was pooled as relative risk (RR) using OpenMeta [Analyst] software for windows. We assessed the heterogeneity using a Chi-square test and its extent was determined by I-square, such that values of p < 0.1 or I2>50% were significant indicators of heterogeneity. We used the random-effects model to analyse heterogeneous data and fixed-effects model for the analysis of homogeneous data, and performed a sensitivity analysis to solve the heterogeneity whenever detected.

## Results

### Literature search

**Figure 1:**
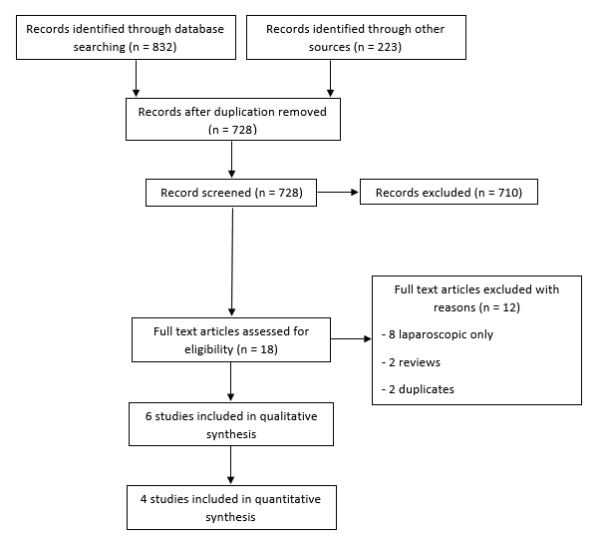
PRISMA flow chart

By searching PubMed, Web of Science, Scopus, and Cochrane Central Register of Controlled Trials (CENTRAL), we identified 832 records. We removed duplicates and the remaining 505 records were screened for eligibility. Only 18 studies were further included for full-text screening. We included four studies from this step. We did not find any missing papers after screening the references of the included trials and previous systematic review (223 references). As a result, we included six studies, with four of them being eligible for meta- analysis. The literature search process is described in a PRISMA flow diagram in Figure 1.

### Characteristics of the included studies

We included one case series study, one cross- sectional, and four retrospective cohort studies with 70 cases of interstitial ectopic pregnancy in the laparoscopic surgery group and 83 cases in the open surgery group. Summary of the included studies and their results are shown in Table I and baseline characteristics of their patients are shown in Table II.

**Table I:** Complete summary of the included studies and their findings (1/2).

Study ID	Study design	Sites and time	Participants and main inclusion criteria	Total cases n	Type of Laparoscopic surgery, (n)	Type of Traditional surgery, (n)	Investigations	Results	Conclusion of the study
Jiang 2018 (32)	Retrospective analysis	Nanjing Drum Tower Hospital, China. Records from July 2010 to December 2015	Patients with intrauterine pregnancy along with feature of a co-existing interstitial pregnancy: A gestational sac visualized high in the fundus. not surrounded by 5 mm of myometrium in all planes.a gestational sac seen separately and < 1 cm from the most lateral edge of the uterine cavity.	17	Laparoscopic cornual resection, (7)	Laparotomy with cornual resection, (3)	Transvaginal ultrasound scan	Compared with laparotomy, laparoscopic cornual section showed shorter operative time (median 40 vs. 70 min), less blood loss (150 vs. 400 ml) and shorter hospital stay (2 vs. 4 days).	Laparoscopic cornual resection is a feasible approach with favorable surgical and long-term pregnancy outcomes.
Hwang 2010 (26)	Retrospective analysis	The Korea University Medical Center, South Korea. Records from January 1998 to October 2009	Patients with interstitial pregnancy who were treated with open cornual resection or laparoscopic cornual resection.	88	Laparoscopic cornual resection, (34)	Open cornual resection, (54)	Transvaginal ultrasound scan	There were no statistically significant differences between the two groups for the mean operation time, estimated blood loss, blood loss of more than 1000 mL, blood transfusion requirements, and complications. The mean number of postoperative hospital days was shorter in the laparoscopy group than in the laparotomy group (4.53 ± 1.44 days versus 5.89 ± 1.86 days, respectively; P < 0.001).	Laparoscopic cornual resection is a safe and less invasive procedure with a reasonable complication rate and shorter hospital stay.
Ghazali 2018 (27)	Retrospective cohort	Putrajaya Hospital, Putrajaya, Malaysia. Records from January 2005 to December 2014,	Patients with interstitial pregnancy who were treated with open cornual resection or laparoscopic cornual resection.	14	Laparoscopic cornuotomy, (7)	Open cornuotomy, (7)	Physical examination, transvaginal sonography, full blood count, and serum human chorionic gonadotrophin (hCG) levels.	The duration of hospitalization and mean operating time were both significantly shorter in the LC group than in the OC group (1.43 ± 0.54 versus 2.57 ± 0.79 and 61.4 ± 15.7 min versus 97.1 ± 38.2 min, respectively, P < 0.05).There were no statistically significant differences between both groups for the estimated blood loss, requirement of blood transfusion, complications, and future fertility.	Laparoscopic cornual resection (cornuotomy) is a safe and less invasive procedure with a comparable complication rate. It has shown that it is feasibility and should be considered as initial treatment in managing those cases in trained hand surgeons.
Sagiv 2013 (22)	Retrospective cohort	Wolfson Medical Center, Holon, and 2Sackler Faculty of Medicine, Tel-Aviv University, Tel-Aviv, Israel. Records from June 1997 to June 2007.	Patients with interstitial pregnancy who were treated with laparotomy, medical treatment with systemic methotrexate, or laparoscopy.	14	Laparoscopy cornuostomy, encircling, or salpingectomy, (8)	Laparotomy, (5)	Transvaginal ultrasound scan	The first four women, with significant hemoperitoneum, were treated by laparotomy. Of the next 10 women, four were selected for medical treatment with methotrexate. Only one case was treated successfully. The other six women had laparoscopic treatment. Of nine laparoscopies, one was converted to laparotomy due to excessive blood loss during the procedure. Of nine women desiring a child, three were infertile, whereas six conceived with an intrauterine pregnancy.	A change from diagnosis later in pregnancy and laparotomy to more conservative treatment, mainly by laparoscopy, suggests a possibly better subsequent pregnancy rate.
Tulandi 2004 (23)	Cross sectional	Cases from 1999 to 2002.	Patients with interstitial pregnancy who were treated with laparotomy, methotrexate, or laparoscopy.	32	Laparoscopy, (11)	Laparotomy, (13)	Transvaginal ultrasound scan and diagnostic laparoscopy and laparotomy.	Persistently elevated serum human chorionic gonadotropin levels were found in one patient after laparoscopic cornual excision, and she was successfully treated with methotrexate. Fourteen cases (43.7%) of rupture of interstitial pregnancy were found. This included five cases (15.6%) of heterotopic pregnancy; all were the results of in vitro fertilization, and all ruptured at the time of diagnosis. Subsequent pregnancy was achieved in ten patients. No uterine rupture was encountered during pregnancy or labor	Ipsilateral salpingectomy, previous ectopic pregnancy, and in vitro fertilization are predisposing factors for interstitial pregnancy. Contrary to previous belief, rupture of interstitial pregnancy occurs relatively early in pregnancy. In selected patients, laparoscopic cornual excision is a viable treatment option.
Warda 2014 (24)	Case series	N/A	Cases of interstitial ectopic pregnancy	4	Laparoscopic cornuostomy and removal of products of conception, (3)	Cornuostomy by laparotomy, (1)	Transvaginal ultrasound scan and diagnostic laparoscopy	Subsequent successful reproductive outcomes are presented.	Progressively conservative surgical measures are being used to treat interstitial pregnancy successfully, with no negative impact on subsequent pregnancies.

**Table II:** Baseline characteristics of enrolled patients in the included studies.

Article ID	Groups	Cases, n	Age, mean (SD)	Gestational age (days), mean (SD)	Symptomatic, number (%)	Ruptured ectopic, number (%)	Parity, mean (SD)	Gravidity, mean (SD)	Ovulation induction number (%)	Sexually transmitted disease, number (%)	Previous laparotomy, number (%)	Previous D & C, number (%)	Previous other tubal pregnancy, number (%)	Previous ectopic pregnancy, number (%)	Previous pelvic surgery, number (%)	Spontaneous conception, number (%)	In vitro fertilization, number (%)	laparoscopic salpingectomy, number (%)	left tubal litigation, number (%)
Jiang 2018 (24)	laparoscopic cornual resection	7	30.71 (3.04)	52.71 (13.78)	4 (57.14)	-	0	4.25 (4.83)	-	-	-	-	-	-	-	-	7 (100)	5 (71.43)	1 (14.29)
	Laparotomy with cornual resection	3	27.67 (2.52)	46.33 (7.51)	1 (33.33)		0	2.33 (2.31)									3 (100)	2 (66.67)	1 (33.33)
Hwang 2010 (30)	laparoscopic cornual resection	34	31.12 (5.99)	55.87 (13.08)	70 (79.5)	8 (23.5)	0.82 (0.9)	-	-	-	34 (38.6)	66 (75.0)	14 (15.9)	1 (1.1)	-	-	1 (1.1)	-	-
	Open cornual resection	54	32.74 (5.11)	54.41 (10.61)		19 (35.2)	0.98 (0.74)												
Ghazali 2018 (31)	Laparoscopic cornuotomy	7	29.3 (5.9)	-	6 (85.7)	5 (71.4)	-	2.9 (0.7)	-	-	-	-	3 (42.9)		4 (57.1)	6 (85.7)	1 (14.3)	-	-
	Open cornuotomy	7	31.4 (7.3)		7 (100)	6 (85.7)		2.7 (1.5)					1 (14.3)		3 (42.9)	7 (100)	0 (0)		
Sagiv 2013 (22)	Laparoscopy	8	34.38 (5.83)	49 (5.29)	3 (21.4)	7 (50)	2.5 (1.2)	4.25 (1.58)	-	-	-	-	5 (35.7)		-	-	-	4 (28.6)	-
	Laparotomy	5	31.8 (6.91)	78.4 (34.79)			1.8 (1.92)	5.2 (2.59)											
Tulandi 2004 (23)	Laparoscopy	11	32.6 (5.66)	37.8 (23.24)	-	5 (45.4)	-	-	1 (3.1)	8 (25.0)	-	-	13 (40.6)		-	-	11 (34.4)	12 (37.5)	
	Laparotomy	13		51.1 (10.08)		9 (69.2)											
Warda 2014 (27)	Laparoscopic cornuostomy and removal of products of conception.	3	32 (3.46)	-	-	-	-	-	-	-	-	-	-	-	-	1 (33.33)	2 (66.67)	1 (33.33)	-
	Cornuostomy by laparotomy	1	36														1 (100)		

### Results of Risk of Bias Assessment

Two cohort studies had a fair quality according to the NIH quality assessment tool for Observational Cohort and Cross-Sectional Studies. The other two cohort studies and the only cross-sectional study were poor quality. The case series study was fair in quality according to the NIH quality assessment tool for case series studies. For more details and answers to all assessment questions in each study, see supplementary Table I for cohort studies and Table II for a case series study.

### Analysis of Outcomes

#### 1. Postoperative hospital stay (days)

**Figure 2:**
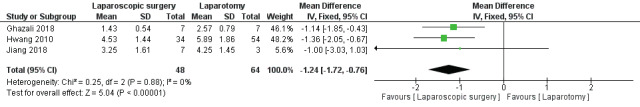
Mean difference (MD) in Postoperative hospital stay.

Three studies reported postoperative hospital stay (days) (Ghazali et al., 2018; Hwang et al., 2011; Jiang et al., 2018). The pooled mean difference (MD) showed that laparoscopic surgery was significantly associated with less hospital stay duration than open surgery (MD = -1.42 days, 95% CI [-1.72, -0.76], P > 0.0001); Fig.2. Pooled results were homogenous (P = 0.88, I2 = 0%).

#### 2. Operation time (minutes)

**Figure 3:**
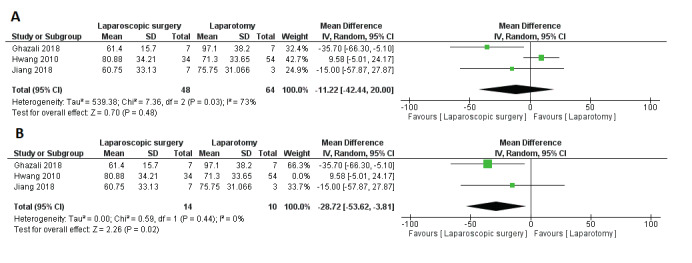
Mean difference (MD) in Operative Time .

Three studies reported operation time (minutes) (Ghazali et al., 2018; Hwang et al., 2011; Jiang et al., 2018). The pooled effect estimate revealed no significant difference between laparoscopic surgery and open surgery in terms of operative time (MD = -11.22 minutes, 95% CI [-42.44, 20], P = 0.48); Fig.3 (A). Pooled results were heterogeneous (P = 0.03, I2 = 73%) and the detected heterogeneity was best resolved after excluding Ghazali et al 2018 (P = 0.29). Homogeneous results also did not favour either group (MD = 5.90, 95% CI [-11.30, 23.09], P = 0.50; Fig.3 (B).

#### 3. Blood loss (ml)

**Figure 4:**
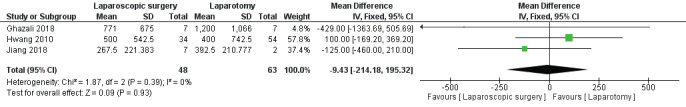
Mean difference (MD) in Blood loss.

Blood loss (ml) was reported by three studies (Ghazali et al., 2018; Hwang et al., 2011; Jiang et al., 2018). The pooled mean difference (MD) showed no significant difference between laparoscopic surgery and open surgery in terms of blood loss (MD = -9.43, 95% CI [-214.18, 195.32], P = 0.93); Fig.4. Pooled results were homogenous (P = 0.39, I2 = 0%).

#### 4. Post- and intraoperative complications

**Figure 5:**
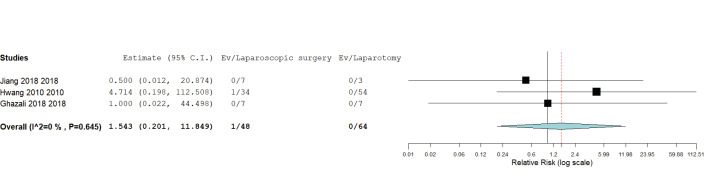
Postoperative and intraoperative complications.

Three studies reported post- and intraoperative complications (Ghazali et al., 2018; Hwang et al., 2011; Jiang et al., 2018). The pooled relative risk (RR) revealed no significant difference between laparoscopic surgery and open surgery in terms of post- and intraoperative complications (RR = 1.54, 95% CI [0.20, 11.85], P = 0.68); Fig.5. Pooled results were homogenous (P = 0.65, I2 = 0%).

#### 5. Need for blood transfusion

**Figure 6:**
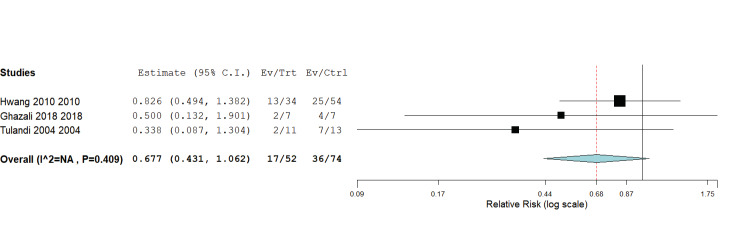
Blood Transfusion.

Two studies reported the need for blood transfusion (Ghazali et al., 2018; Hwang et al., 2011; Jiang et al., 2018). The pooled mean difference (MD) showed no significant difference between laparoscopic surgery and open surgery in terms of need for blood transfusion (RR = 0.68, 95% CI [0.43, 1.062], P = 0.09); Fig.6. Pooled results were homogenous (P = 0.41, I^2^ = 0%).

### Miscellaneous outcomes

For pregnancy outcome as reported by Sagiv et. al (2013), three out of five women undergoing laparotomy became infertile, one underwent a tubal ligation, and only one woman became pregnant and delivered by cesarean section. But out of eight women managed by laparoscopy, three women were undesirable for pregnancy, one outcome was not reported, one had an early miscarriage, and three became pregnant and delivered by cesarean section.

Tulandi et al. (2004) reported that in patients managed by laparoscopy, the hemoperitoneum encountered was 1385.7 ± 978.8 mL in the laparotomy group and 460.0 ± 70.7 mL in the laparoscopy group. In the laparotomy group, no patient needed any subsequent treatment but in the laparoscopy group, the first treatment failed in one patient and needed methotrexate as a subsequent treatment.

Warda et. al (2014) presented four cases of interstitial pregnancy; the first case was a 36-year-old woman treated with cornuostomy by laparotomy and then received a third intracytoplasmic sperm injection cycle, subsequently delivering an intact female after a pregnancy period devoid of any complications. The other three cases ( two aged 30, 30, and one 36 ) were treated with laparoscopic cornuostomy and removal of products of conception. They also underwent another in-vitro fertilisation cycle, then delivering without any complications and with no adverse neonatal outcomes.

## Discussion

We found that laparoscopic surgery was significantly associated with less postoperative hospital stay period and less operation time than open surgery. However, we found no difference between both types in terms of blood loss, post- and intraoperative complications, and need for blood transfusion.

Our results were consistent with other studies published in the literature. Laparoscopic surgery has many advantages such as minimal skin incision and a shorter hospital stay period, preserves the uterus for future fertility, and is associated with improved and fast recovery and less postoperative pain (Soriano et al., 2008; Ng et al., 2009), but also has some minor disadvantages such as hematomas of the abdominal wall occurring near the incisions, some abdominal or pelvic infections, although serious laparoscopy complications are rare (Rempen et al. 1999).

Laparotomy is the second line of management when there is no laparoscopic expertise or when adequate closure or hemostasis cannot be achieved by laparoscopic surgery. However, it has a lot of risks, ranging from the general risks of anesthesia and surgery to incisional hernia, infections, bleeding, and injury of pelvic or abdominal organs. Also, it is associated with longer hospital stay periods as reported in some studies (Hwang et al., 2011; Ghazali et al., 2018).

Two previous systematic reviews have described a roadmap for nearly all medical and surgical options, and both recommended using laparoscopic surgery in most conditions (Moawad et al., 2010; Cucinella et al., 2014).

A previous meta-analysis compared laparoscopy against laparotomy for ectopic pregnancy and concluded that laparoscopy is better than laparotomy. However, contrary to our results, they found no difference between laparoscopy and laparotomy in terms of operation time, which we found was lower in the laparoscopic group. Additionally, they found that intraoperative blood loss was lower in the laparoscopy arm compared with laparotomy (Gao et al., 2009).

Some studies compared different laparoscopic approaches; the study by Lee et al. (2016) compared laparoscopic cornual resection and laparoscopic cornuotomy, and found no significant difference between them in haemoglobin levels after the operation, persistent interstitial pregnancy and incidence of major complications, though the operation time was significantly shorter for cornuotomy than that for cornual resection. Also, Gasparria et al. (2018) compared conventional versus single port laparoscopy, and found no difference between them in terms of haemoglobin levels, need for blood transfusions, operation time, length of hospital stay period, and post- and intra- operative complications.

In a cost-effectiveness comparison between laparoscopic and laparotomy, Gray et. al in 1995 showed that at lower costs, laparoscopy produced final outcomes comparable to those of laparotomy. Also, Ghazali et. al (2018) stated that laparoscopy was associated with financial savings.

We included all studies comparing laparoscopic surgery with laparotomy in interstitial ectopic pregnancy patients. Additionally, most of our results were homogenous and we managed to solve the heterogeneity detected among studies.

However, we have some limitations in our study such as: 1- Small number of the included studies and small sample size with the absence of clinical trials; 2- lack of data about long term effects; 3- all of the included studies were observational which are considered a low level of evidence; 4- we only included English studies; only three studies with 112 cases were eligible for analysis, which might have an influence on our results in situations where the laparoscopic approach is not applicable.

## Conclusion

Our analysis shows that in women with interstitial ectopic pregnancy, management with laparoscopic surgery is associated with reduced postoperative hospital stay duration. There was no difference in terms of operation time, blood loss, post- and intraoperative complications, and need for blood transfusion.

Further studies, especially interventional studies with longer follow up duration and larger sample sizes, are needed to produce more valid results and until that we recommend using laparoscopic surgery if available as it has some advantages over open surgery.
